# To Debride or Not to Debride: A Review of Wound Management for Stevens-Johnson Syndrome and Toxic Epidermal Necrolysis

**DOI:** 10.7759/cureus.55350

**Published:** 2024-03-01

**Authors:** Christina D Enescu, Adam J Elder, Hany Deirawan, Meena Moossavi

**Affiliations:** 1 Department of Dermatology, Wayne State University School of Medicine, Detroit, USA; 2 Department of Medical Education, Wayne State University School of Medicine, Detroit, USA

**Keywords:** conservative management, surgical debridement, wound care, burn care, toxic epidermal necrolysis, stevens-johnson syndrome, general dermatology

## Abstract

Stevens-Johnson syndrome (SJS) and toxic epidermal necrolysis (TEN) are immune-mediated skin reactions with high mortality as a result of severely compromised skin barrier function. Currently, there is no consensus on the topical management of these conditions. Some advocate for surgical debridement of affected skin as a means of preventing infection and facilitating reepithelialization with synthetic and biological wound coverage. Others prefer a conservative approach that relies on leaving the blistered skin in situ. A consensus is lacking, primarily due to the rarity of the disease and the lack of high-quality evidence supporting one particular form of management. The goal of this review is to explore and compare the two treatment approaches for SJS and TEN, namely conservative management and surgical debridement.

## Introduction and background

Stevens-Johnson syndrome (SJS) and toxic epidermal necrolysis (TEN) comprise a spectrum of severe mucocutaneous reactions characterized by epidermal necrosis and detachment, usually triggered by an immune response to medication [1]. They are clinically similar and mainly differ in body surface area distribution. Involvement of <10% of total body surface area (TBSA) is classified as SJS, whereas >30% of TBSA involvement is considered TEN. Involvement of 10% to 30% TBSA is classified as SJS/TEN overlap (SJS/TEN). These are rare conditions, with an incidence of 1.0 to 6.0 per million for SJS and 0.4 to 1.2 per million for TEN [2]. The overall mortality rate is around 30%, with up to 10% for SJS and 50% for TEN. Medications are the leading cause of both SJS and TEN (SJS-TEN). The most common culprits include allopurinol, aromatic antiepileptic drugs, lamotrigine, antibacterial sulfonamides, nevirapine, and nonsteroidal anti-inflammatory drugs [1].

The prodrome consists of fever and flu-like symptoms, followed by the development of mucocutaneous lesions one to three days later [1]. Skin lesions typically start with diffuse erythema or erythematous, coalescing macules with purpuric centers. Progression leads to bullae and vesicles, followed by skin sloughing. Nikolsky’s sign, or superficial sloughing following gentle pressure on the skin surface, can be observed [1].

In SJS-TEN, epidermal detachment occurs at the epidermal-dermal junction, but the dermis and its collagen and reticular fibers are preserved [3]. Epithelial remnants present in hair follicles and sweat glands facilitate epithelial regeneration. Because the dermis is preserved in SJS-TEN, there is less scarring after healing than in second-degree burns. Protecting the dermis from infection, shearing, and injury during healing is critical to minimizing scarring [4-6]. Proper wound care, careful dressing changes, and adequate wound conditions, like sufficient moisture levels, are essential for maintaining a healthy dermis and preventing scarring, infection, heat loss, and dehydration [7].

Restoration of skin barrier function is a multifaceted process that aims to regenerate a healthy epidermis. In the acute phase, the cessation of further damage to the epidermis by aborting the source of immune dysregulation is essential. Other elements of wound care and treatment have been subject to substantial advances and continued research beyond the focus of this review. Beyond hemodynamic and supportive care, management is focused on temporary compensation for the damaged epidermal barrier, infection control, and the facilitation of epidermal regeneration.

Currently, there is no consensus regarding optimal wound care [8]. Some centers favor surgical debridement, while others prefer a conservative approach that relies on anti-shear measures, leaving the detached skin in place. Some support a middle approach with early removal of the devitalized epidermis without aggressive wound debridement [9]. Both surgical debridement and conservative anti-shear wound care have shown equivalent rates of reepithelialization [8], although there have not been any clinical trials to compare the two approaches directly. The gaps in knowledge of the mechanism of SJS-TEN disease progression and the lack of formal randomized control trials due to the rarity of the condition make clinical management non-standardized and controversial.

## Review

Conservative approach

With conservative management, the detached epidermis is left in situ. It acts as a split-thickness skin graft or biologic dressing to facilitate wound healing and protect the dermal layer underneath [10,11], allowing the epidermis to form a barrier against desiccation and infection. This approach aids in preserving the viable dermis and dermal appendages responsible for reepithelialization. Observational data from one study indicated that if detached skin was left in place, it acted as a skin graft and resulted in less morbidity, mortality, and depigmentation after healing, leading this institution to transition from debridement to anti-shear therapy [12].

Aspiration of blister fluid is recommended to allow the detached epithelial layer to settle on top of the dermis, aiding its function as a topical biologic dressing and aiding the surface for proper healing [12,13]. Blisters should also undergo aspiration to avoid extension of epidermal separation at the level of the intercellular junctions [11].

Removing cytokines and other signaling molecules may help reduce the propagation of the inflammatory process. One hypothesis regarding the biological mechanism of SJS-TEN is that cytokines within blister fluid led to the recruitment of cytotoxic lymphocytes to the epidermis and the upregulation of Fas ligand (FasL), both responsible for cell apoptosis [13,14]. Analysis of blister fluid composition from patients with SJS-TEN has revealed high concentrations of secretory granulysin, FasL, TNF-α, perforin, and granzyme B [15-17] along with cytotoxic CD8+ T cells and natural killer-like cytotoxic T cells [16,18]. At the dermo-epidermal zone, high concentrations of CD14+ cells may increase CD8+ T cell proliferation and cytotoxicity [16,19]. Thus, aspiration of the blister fluid may theoretically help control the spread of blistering [12,13]. Early debridement of the denuded epidermis to remove the blister fluid is a more aggressive alternative.

It has been proposed that SJS-TEN should be managed less aggressively than burns because burn wounds are characterized by complete necrosis of the epidermal layers. At the same time, the damage in SJS-TEN occurs between viable layers of skin [11]. Some epidermal cells in SJS-TEN are detached but not directly injured by the inflammatory process. Thus, layers of skin may still retain biological function, so epidermal remnants may have some benefit if left in situ. One study explains that although the epidermis exfoliates from the dermis in SJS-TEN, the basal layer of the epidermis is well perfused, and there are remnants of basal keratinocytes on the skin appendices [20,21]; therefore, a conservative approach should be executed to avoid damage to this layer as it may aid reepithelialization.

Topical emollients and dressings help maintain barrier function, limit fluid and heat loss, prevent adhesion to other surfaces, aid reepithelialization, and prevent infection [10,22]. Collagen dressings like Biobrane® limit fluid loss and aid reepithelialization due to fewer dressing changes and ease of application [11]. Some studies favoring a conservative approach argue that biological dressings like Biobrane®, cadaveric allograft, or porcine allograft are still good to apply to affected areas even with the skin left in situ [11,22]. In other words, debridement may not be necessary before applying biological dressings to wound surfaces.

A potential downside of the conservative approach is the risk of bacterial colonization of sloughed skin, as a necrotic epidermis serves as a substrate for bacteria like *Staphylococcus aureus* and *Pseudomonas aeruginosa* [10]. Despite this, an Internet survey showed that most clinicians (67%) preferred a conservative approach [23]. The conservative approach is beneficial if transfer to a burn center for more aggressive surgical management is not immediately available [10]. A conservative protocol also reduces the cost and pain associated with operative debridement and dressing changes. With less manipulation of the skin via debridement and dressing changes on exposed, denuded skin, the patients are subjected to less overall pain during treatment.

Several studies have found positive outcomes with conservative management, such as a lower mortality rate than with aggressive management [24], less pain for patients [20], and successful skin healing with no adverse reactions or need for surgical intervention (Table 1) [25,26]. In contrast, multiple studies have also reported unchanged or unfavorable outcomes [1,7,20,26-29]. In one study, there was a lower mortality than that predicted by the severity-of-illness assessment score for SJS-TEN (SCORTEN) [29], but this was not statistically significant. In another study, the mortality rate with a conservative protocol was only 1% lower than that of a previously used surgical protocol [29]. Although these studies suggest greater patient comfort and ease of management with conservative treatment, no evidence indicates that mortality outcomes are superior to surgical management. Limitations of these studies include small sample sizes, some involving less than 30 patients [13,20,25,26,29], and a lack of comparison groups due to the rare nature of the condition and urgency of treatment [7,13,20,25-28,30]. Only one study was noted to have a comparison group [29]. These studies are also mostly retrospective reviews, which provide weaker evidence than controlled clinical trials. Refer to the Appendices section for a clearer understanding of the levels of evidence mentioned in Table 1.

**Table 1:** Summary of studies favoring conservative management SCORTEN: severity-of-illness score for SJS-TEN, SJS: Stevens-Johnson syndrome, TEN: toxic epidermal necrolysis; TBSA: total body surface area

Author (year of study)	Study objective	Type of study	Number of patients	Study treatment protocol/methods used	Study findings	Level of evidence
Papp et al. [7](2018)	Compare the actual outcome of patients with the predicted outcome (SCORTEN score)	10-year retrospective review and literature review	67 patients with SJS or TEN	Blister debridement was done on only 44% of patients with TEN, 14% with SJS/TEN, and 7% with SJS, while the rest were managed conservatively	Mortality was 20.9%, the highest rate in the TEN group (35%). The most common cause of death was an infection	4
Dorafshar et al. [12](2008)	Compare the actual outcome of patients with the predicted outcome (SCORTEN score)	19-year retrospective review	48 patients with SJS or TEN	Patients were treated with a conservative, anti-shear wound care protocol	The mortality rate was 27%, an 11% reduction compared to the SCORTEN score (not statistically significant, likely due to the small sample size)	4
Zajicek et al. [20] (2011)	To present experience with treating SJS-TEN at a burn center	10-year retrospective review	22 patients with SJS or SJS or TEN	The patients were treated conservatively by leaving the detached epidermis in place and covering the affected areas with topical materials	Mortality was 32%. Wound infection occurred in 31% of patients. With no wound infections, skin drying, or mechanical traumas, skin reepithelialization occurred in three weeks	4
Stella et al. [24] (2007)	Compare previous five-year experience with prior aggressive wound management with recently adopted six-year conservative management	Retrospective review	Eight patients with TEN were treated aggressively; 23 patients with SJS or TEN were treated conservatively	Conservative wound care consisted of blister exudate evacuation, detached epidermis replacement, and remaining epidermis preservation	Mortality with conservative management (74%) was lower than with aggressive debridement (75%)	2A
Dalli et al. [25] (2007)	Document patient demographics, causative agents, TBSA involved, complications, treatment, and outcome	Five-year retrospective review	16 patients with SJS or TEN	Fluid within blisters and bullae was aspirated, and the viable epidermis was reapplied to the dermis. Paraffin emollients were used. Biobrane®, allograft, or xenograft with Mepitel® or Jelonet® and butadiene-soaked gauze were also used	Excellent results and successful skin healing with conservative wound care and the use of nanocrystalline silver-impregnated gauze. No adverse reactions and no need for surgical intervention	4
Rajaratnam et al. [26](2010)	Document the etiology, clinical features, complications, and outcomes of patients. Evaluate the effects of treatment	12-year retrospective review	21 patients with SJS or TEN	Blisters were punctured but not deroofed. Dressings were applied to detached skin to prevent mechanical disruption	Mortality was 38%. Conservative management had successful healing in 18 patients. Only three patients suffered from long-term skin complications, including thinning of scalp hair, loss of fingernails, and irregular skin pigmentation	4
de Prost et al. [27] (2010)	Describe the epidemiology, early predictors, and predictive value of bloodstream infections in SJS or TEN management	11-year retrospective cohort study	179 patients with SJS or TEN	Patients were treated conservatively with daily antiseptic baths or pulverization, dressings, and no aggressive debridement	This resulted in 48 patients (26.8%) with bloodstream infections. Several variables contributed to this outcome, including age >40 and TBSA percentage of detached skin >30%	4
Firoz et al. [28] (2012)	Compare the actual outcome of patients with the predicted outcome (SCORTEN score)	Five-year retrospective review	82 patients with SJS or TEN	The wounds were treated conservatively and wrapped with Kerlix gauze soaked with 0.5% silver nitrate solution	Mortality was 29%, with SCORTEN scores accurately predicting mortality	4

Surgical approach

Surgical management involves debriding the detached epidermis and applying biosynthetic dressings, allografts, or xenografts for physiological wound closure [10]. Removing the denuded epidermis, which can serve as a nidus for infection, can limit cutaneous infections that impair reepithelialization or lead to sepsis. Reducing the bacterial count on the wound surface is also critical for proper wound healing, skin grafting, and minimized scarring [6]. Debridement is essential for achieving a clean wound bed for healing, preventing wound infection, and providing a clean surface for biological dressing application [31]. Wound debridement also potentially leads to faster wound healing than conservative management [6,32,33].

Some argue that surgical management should be the standard treatment for SJS-TEN as this is a “burn-like” condition and, therefore, necessitates aggressive treatment to avoid burn-associated complications like infection [13]. Given that cytokines in blister fluid may recruit cytotoxic T cells to the epidermis and lead to further cell apoptosis [13,15,16,31], early removal of the blistered epidermis and blister fluid may help limit disease progression.

Debridement of blisters in SJS-TEN is considered an aggressive approach, usually reserved for patients with greater than 30% TBSA of epidermal detachment, hence TEN, or who have failed conservative management [10]. This approach may also be considered if the disease is not well controlled or blistering continues to spread.

A lower bioburden of the affected tissue has been shown to accelerate wound healing through experiments [34]. Poor wound healing is mainly caused by wound infection because bacteria interfere with wound healing by secreting harmful toxins, enzymes, and waste products into the wound and disrupting collagen formation and organization [33]. Bacterial exotoxins can interfere with collagen remodeling by impairing collagen synthesis [33]. Bacterial proteases may alter the activity of matrix metalloproteinases, causing the breakdown of the extracellular matrix and tissue destruction [33]. Bacteria also lead to local hypoxia, damaging cells necessary for collagen production and immune function [33]. Cleansing the affected wound area through more aggressive surgical debridement may be necessary to reduce the bacteria in the wound bed and prevent insufficient wound healing [33].

Surgical management must be careful not to damage the underlying dermis, as damage to or loss of dermal tissue leads to scarring and poor wound healing [5]. Debridement is now commonly done with hydrosurgery, as it is more gentle than blunt or sharp debridement [5]. The saline lavage of hydrosurgery systems cleans the wound without injuring the healthy dermis [13]. Copious lavage reduces bacterial load, providing a clean surface for biological dressings like Biobrane® [31], which may optimize the benefits of biological dressings [6].

High-pressure parallel waterjets employ a high-velocity, high-pressure stream of sterile saline that runs parallel to the skin surface and tangentially excises tissue [6]. Multiple studies indicate superior performance in debriding contaminated wounds with hydrosurgery over conventional techniques [6,35-37]. Versajet is the prototype for this technique and is probably the most used hydrosurgery device currently. Other hydrosurgery techniques include traditional pulse lavage, which utilizes an external suction force [33]. Traditional pulse lavage and Versajet were equally effective for removing bacteria and debris from wound beds [33].

Hydrosurgery works best when the tissue being removed is softer than the underlying tissue left behind, making it a practical and more gentle mechanism of debridement for SJS-TEN patients [6]. The ability to reach small crevasses within the dermis for optimal cleansing is another advantage of this technique compared to blunt debridement [31]. For example, Versajet can debride contoured and challenging areas, like the face, hand, and foot [6]. This precision allows the sparing of critical dermal appendages needed for the proper formation and migration of keratinocytes and, thereby, successful skin reepithelialization with minimal scarring [6,38].

Given that the epidermis is already detached from the underlying dermis in SJS-TEN, aggressive debridement and Versajet may not be necessary if removal of detached skin is desired [25,39]. Instead, a milder approach with gentle mechanical washing of denuded skin can be made, perhaps with a water bath or washcloth instead of the high-pressure stream used in Versajet [39].

Enzymatic debriding may be a gentler method of debriding the skin without surgery. Applying enzymatic debriding ointment after gentle cleansing and removal of nonviable tissue showed positive outcomes in one study, as there were no documented cases of secondary infection [40].

Surgical management has associated risks. Aggressive wound debridement may not be preferable, as this can damage the underlying dermis and healthy tissue [41]. Sharp surgical debridement generally should be avoided for this reason [3]. Although most bloodstream infections in SJS-TEN patients originate from the skin, surgical debridement and skin grafting are performed under general anesthesia and require mechanical ventilation, which can expose the patient to other nosocomial infections like ventilator-associated pneumonia and urinary tract infections [42].

Several studies have reported improved survival outcomes compared with the SCORTEN predicted outcomes [1] with the debridement approach using gauze [43], Versajet [13], or unspecified surgical techniques (Table 2) [41]. Studies also noted facilitated healing and reduced pain and moisture loss [44], primarily due to artificial skin substitutes used with surgical debridement. One study also reported no long-term wound scarring at follow-up [45]. However, using the SCORTEN to demonstrate efficacy is a significant limitation given its tendency to overestimate mortality, at least in centers using standardized protocols [40]. Limitations of these studies include a small sample size, as some had less than 20 patients [41,43] and a lack of comparison groups [13,40,41,43-45]. Refer to the Appendices section for a clearer understanding of the levels of evidence mentioned in Table 2.

**Table 2:** A summary of studies favoring surgical management SCORTEN: severity-of-illness score for SJS-TEN, SJS: Stevens-Johnson syndrome, TEN: toxic epidermal necrolysis, N/A: not available

Author (year of study)	Study objective	Type of study	Number of patients	Study treatment protocol/methods used	Study findings	Level of evidence
Nizamoglu et al. [13] (2017)	Compare the actual outcome of patients with the predicted outcome (SCORTEN score)	12-year retrospective review	42 patients (32 with TEN, 10 with SJS/TE overlap)	Removed loose or necrotic epidermis and cleaned wounds with a topical antimicrobial agent. For delayed presentation, Versajet was used. Most wounds were covered by Biobrane®, allograft, or xenograft, except in cases with infection or delayed presentation	The mortality rate was 9.52%. The outcome of 42 patients who underwent surgical debridement was lower than predicted by the SCORTEN score	4
Edwards et al. [40](2009)	Summarize the topical wound management of three patients with SJS or TEN	Case series	Three patients with SJS or TEN	Gentle debridement protocol with hydrotherapy, followed by application of an enzymatic debriding ointment. Nanocrystalline silver-coated dressings were also used. In one case, an oat β-glucan cream was used	There were no documented cases of secondary infection	4
McCullough et al. [41](2017)	Compare the actual outcome of patients with the predicted outcome (SCORTEN score)	15-year retrospective review	40 patients with SJS or TEN	Patients underwent wound debridement. The wounds were covered with an antibacterial, silver-releasing dressing. The antimicrobial dressings successfully healed 39 patients	The mortality rate was 10%. The outcome of 40 patients treated with surgical debridement was lower than predicted by the SCORTEN score	4
Zhang et al. [43](2019)	Compare the actual outcome of patients with the predicted outcome (SCORTEN score)	10-year retrospective review	13 patients with SJS or TEN	Gentle surgical debridement with gauze, followed by application of porcine xenograft	The mortality rate of 13 patients treated with surgical debridement (12.5%) was lower than predicted by the SCORTEN score (25.2%)	4
Lim et al. [44] (2016)	Analyze the diagnostic and prognostic value of variables collected on referred SJS-TEN patients	11-year retrospective review	76 patients with SJS or TEN	Surgical debridement of devitalized epithelium followed by application of skin substitutes like porcine xenograft or Biobrane®	Their method was found to facilitate healing and reduce pain and moisture loss	4
Cartotto et al. [45](2017)	Review all aspects of SJS-TEN with a primary focus on management	Literature review	N/A	Aggressive debridement followed by Biobrane®	No long-term wound scarring was observed at follow-up except for punctate scars where staples were inserted	5

## Conclusions

The approach to wound care for patients with SJS-TEN is highly variable and institution-dependent. This has been due to the lack of high-quality clinical evidence and consensus among medical experts. Skin treatment generally follows recent trends in burn wound care. The outcome of SJS-TEN has improved with the evolution of supportive care in specialized centers and advancements in topical therapies and wound coverage material. Due to the rarity and urgency of the condition, no clinical trials have been done to compare different treatment modalities and establish a standardized protocol. Advocates of a conservative approach need strong evidence for its utility, safety, and cost efficiency. This method is comfortable for patients, easy for caretakers, and avoids the added physiologic stress of aggressive surgical treatment. However, preventing infection with conservative wound management is more challenging. Surgical debridement has a role in managing infected skin or lesions at increased risk of infection, such as delayed presentation or refractory and intractable disease. The authors believe that the routine use of more aggressive treatment with surgical debridement lacks convincing evidence of its superiority.

Conservative management and surgical debridement have not been compared in well-designed clinical trials. In the absence of solid evidence for a preferred approach, the authors of this review support a middle approach in which elements of surgical and conservative methods are used and dictated by the disease extent and patient status. Cleansing the skin with a gentle mechanical wash rather than Versajet or surgical debridement is sufficient given SJS-TEN pathology. The combination of gentle debridement of large detached epidermal segments, aspiration of bulla fluid, and anti-shear measures with adjunct immunomodulatory therapies has been the preferred approach for most cases. Aggressive debridement with wound lavage and biological dressings have been the preferred approaches for infected wounds and cases of delayed presentation. Future clinical trials are critically needed to examine some of the most urgent questions in SJS-TEN management. Of particular interest, in the authors’ opinion, is determining the role and safety of surgical management versus maximal medical and supportive therapies in cases of rapid progressive or intractable disease and high-risk surgical candidates with severe disease and multiple medical comorbidities.

## Appendices

**Table 3:** Level of evidence for therapeutic studies referenced in Tables 1-2

Level of evidence [46]	Type of evidence utilized in the study
1A	Systemic review of randomized-controlled trials
1B	Individual randomized-controlled trial (with narrow confidence interval)
1C	All or no study
2A	Systemic review of cohort studies
2B	Individual cohort study (including low-quality randomized-controlled trial)
2C	“Outcomes” research, ecological studies
3A	Systemic review of case-control studies
3B	Individual case-control study
4	Case series (as well as poor-quality cohort studies and case-control studies)
5	Expert opinion without explicit critical appraisal or based on physiology bench research or “first principles”
